# Paracetamol versus Paracetamol Plus Ondansetron on Acute Postoperative Pain

**DOI:** 10.1055/s-0041-1735899

**Published:** 2021-12-15

**Authors:** Mohamad Aryafar, Mahnaz Narimani Zamanabadi, Kourosh Farazmehr, Giti Dehghanmanshadi, Sepideh Davoodinejad, Farshid Gholami

**Affiliations:** 1 Department of Anesthesiology, Faculty of Medicine, Tehran Medical Sciences, Islamic Azad University, Tehran, Iran; 2 Student of Research Committee, Tehran Medical Sciences, Islamic Azad University, Tehran, Iran

**Keywords:** postoperative pain, ondansetron, paracetamol, abdominal surgeries, analgesic use, severity

## Abstract

This study was performed to determine the comparative efficacy of paracetamol alone versus paracetamol plus ondansetron on acute postoperative pain after abdominal surgeries in Azad University hospitals in 2017 and 2019. In this randomized clinical trial, 62 consecutive patients under abdominal surgeries, were randomly divided into two groups, group 1 patient who received paracetamol alone 1 gram and group 2 patient who received paracetamol 1 gram plus 4 mg ondansetron and the pain severities were determined and compared between groups at recovery and after 4 and 24 hours. The results of this study revealed that there were no statistically significant differences between two groups for the postoperative pain severity and analgesic use ( *p* > 0.05). It may be concluded that addition of ondansetron to paracetamol would not result in further postoperative pain reduction and additive use of this drug is not recommended.

Pain is one of the problems that always harms the person and has many unwanted side effects. According to the definition of the International Association for the Study of Pain (IASP), pain is an unpleasant emotional and sensory experience with potential or actual tissue damage. 1 Meanwhile, 2 one of the worst pain that humans endure is acute postoperative pain and the pain is more severe, it creates hemodynamic and metabolic responses to patients. 3 4 About 21% of patients' experience moderate to severe pain after surgery. 5 Insufficient relief of postoperative pain leads to complications such as long recovery, 6 long admission, increased hospital costs, and reduced patient satisfaction. 7 Effective management of postoperative pain is now part of the surgical process and not only reduces the suffering of the patient but also reduces mortality and promotes rapid improvement and early discharge from the hospital, 8 improves the patient's quality of life, and reduces costs. 9 Effective management of postoperative pain involves a multimodal approach in which different drugs are used with different mechanisms and methods of administration. 10 Today, there are various treatments for reducing postoperative pain, each with its own effectiveness and success, 11 which include prescribing synthetic drugs and opioid drugs before or during surgery. 12 Opioids are dose-dependent, with side effects such as nausea and vomiting, itching, urinary retention, respiratory depression, and consequently increased hospitalization time. 13 But the use of nonopioid analgesics reduces side effects. 14 Long-term administration with high doses of analgesics may cause bleeding at the site of gastrointestinal bleeding and renal failure, and nonopioid analgesics do not have these complications. 15 16 Accordingly, in this study, we compared the effect of paracetamol alone or paracetamol plus endonestrone one on acute pain after abdominal surgery in hospitals of Islamic Azad University in 2017 and 2019.

## Methods

This study was a randomized clinical trial study in patients undergoing elective abdominal surgery. The inclusion criteria for this study were elective surgery, individual consent for participation in the study, no history of paracetamol and ondansetron susceptibility, age 20 to 60 years, body mass index (BMI) 20 to 30, tendency to general anesthesia, grade 1 and 2 American Society of Anesthesiologists (ASA), and no history of drug addiction. The exclusion criteria for this study were history of surgery for more than three hours, lack of patient collaboration due to postoperative delirium, complication of surgery, and use of postoperative supplements. After obtaining written consent from patients who underwent elective abdominal surgery and had ASA Classes 1 and 2, they were randomly divided into two groups: group 1: patient who received paracetamol alone 1 gram and group 2: patient who received paracetamol 1 gram plus 4 mg ondansetron. The patients were unaware regarding the type of intervention.

In both groups after induction with fentanyl 2 µg/kg, midazolam 0.05 mg/kg, atracurium 0.5 mg/kg, each hour 0.5 to 1 µg/kg fentanyl and the patients were intubated using tracheal tube (tracheal tube of 7 or 7.5 for the female and tracheal tube 7.5 or 8 for men). To maintain anesthesia, oxygen and nitroxide were used in equal ratio (50% each) plus propofol 6 to 12 mg/kg/h. Each hour 0.001 mg/kg fentanyl and 0.2 mg/kg atracurium were administered to patients.

At the end of the surgery, muscle relaxant drugs were reversed with neostigmine 0.04 mg/kg and atropine 0.02 mg/kg. Extubation of tracheal tubes was taken after returning gag reflux to normal situation.

It should be noted that in the first group, 1 gram of paracetamol was injected intravenously within 20 minutes for patients at the end of the operation. In the second group, ondansetron was given 4 mg and 30 minutes before the end of the intravenous operation, and at the end of the operation, 1 gram of paracetamol was injected intravenously and administered in 20 minutes to patients.

Patients' pain was then assessed by a trained nurse at the time of recovery, 4 hours and 24 hours after the operation, and recorded in the questionnaire. Additionally, the opioid drug was recorded in the questionnaire within 24 hours of operation, and then the two groups were compared by statistical analysis. Collected data was evaluated using checklist and field survey. The clinician responsible for collecting the data was oblivious regarding the type of intervention till the end of the study.

Data were analyzed using SPSS software version 13. For quantitative data, mean, and standard deviations were recorded. Absolute and relative frequency were recorded for qualitative variables. The tests were Chi-square, T-independent, and Fisher, and the level of significance was <0.05.

T-test of two independent samples was used to evaluate the effect of adding ondansetron to paracetamol to improve postoperative pain. Power analysis was used to evaluate the adequacy of the sample size. Power analysis was performed using pwr package. 17 under R software under two different conditions ( Supplementary Figs. S1 and S2 [available online only]). Using significance level 0.1, the results showed that with a sample size of 62 people (31 people with paracetamol plus ondansetron and 31 people with paracetamol) with appropriate power, moderate to high pain relief in the group paracetamol plus ondansetron can test the outcomes of paracetamol. Using significance level 0.05, a sample size of 62 people can be used to compare the pain between two groups with a large effect size. 18

This study was approved by the Research Ethics Committee Board at the (Tehran Medical Sciences, Islamic Azad University, Tehran, Iran) and was in accordance with the Helsinki principles.

## Results

The distribution of age, gender, BMI, ASA class, and type of surgery were identical in two groups ( *p* -values were as follows: *p* = 0.872, *p* = 0.793, *p* = 0.855, *p* = 0.199, and *p* = 0.316, respectively).

**Fig. 1:**
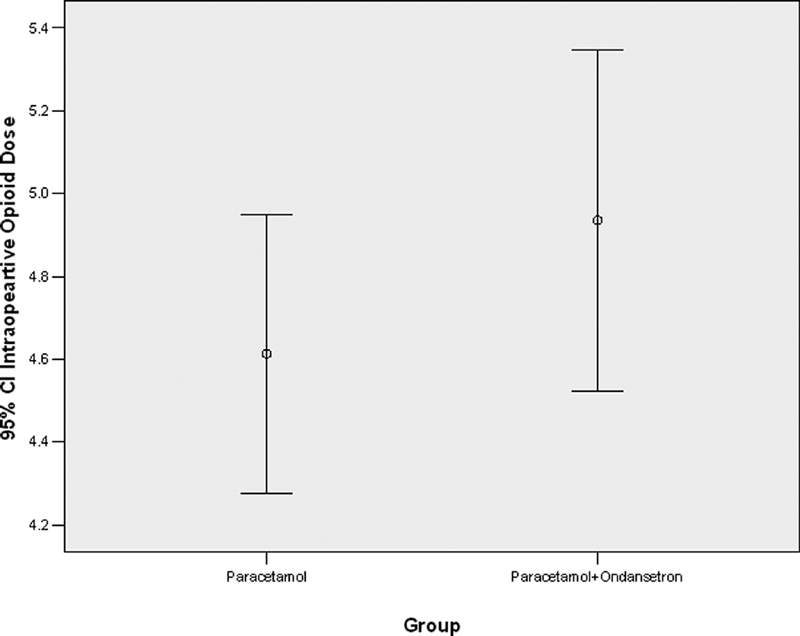
The intraoperative opioid dose in patients receiving paracetamol alone and those who received paracetamol with ondansetron.

**Fig. 2:**
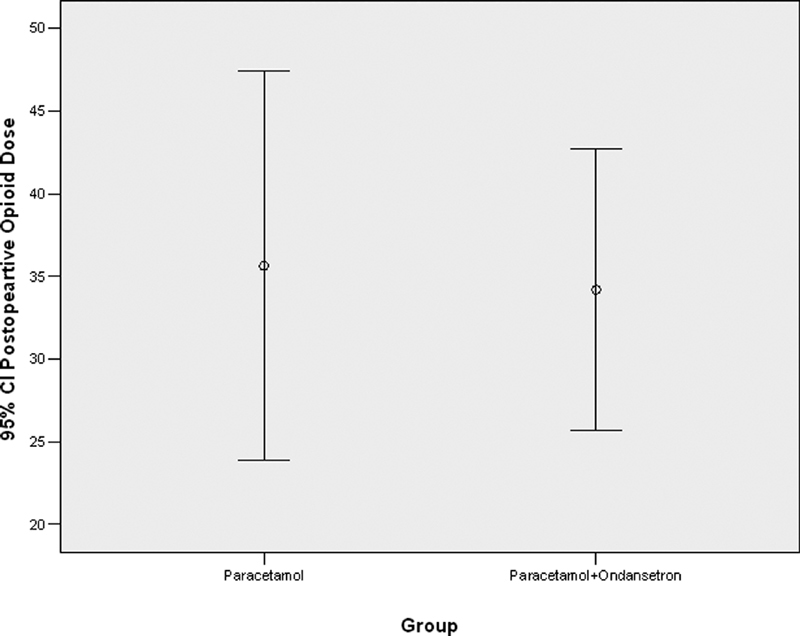
The postoperative opioid dose in patients receiving paracetamol alone and those who received paracetamol with ondansetron.

There was no statistically significant difference between the two groups in the number of opioids administered intraoperatively ( *p* = 0.221). Fig. 1 shows the intraoperative opioid dose in patients receiving paracetamol alone and those who received paracetamol with ondansetron. Also, there was no statistically significant difference between the two groups in the postoperative drug dose ( *p* = 0.839). Fig. 2 shows the postoperative opioid dose in patients receiving paracetamol alone and those who received paracetamol with ondansetron.

**Fig. 3:**
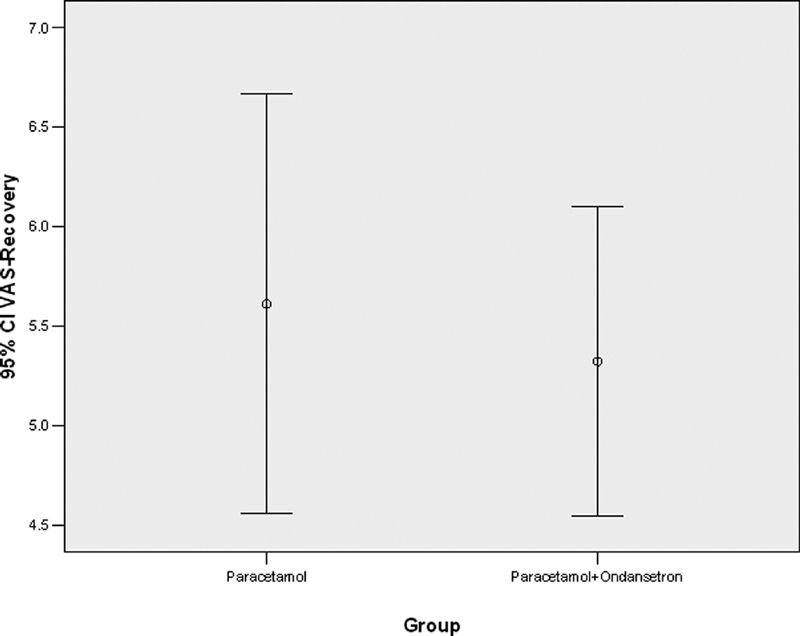
Showing the severity of pain at the time of recovery in patients receiving paracetamol alone and those who received paracetamol with ondansetron.

**Fig. 4:**
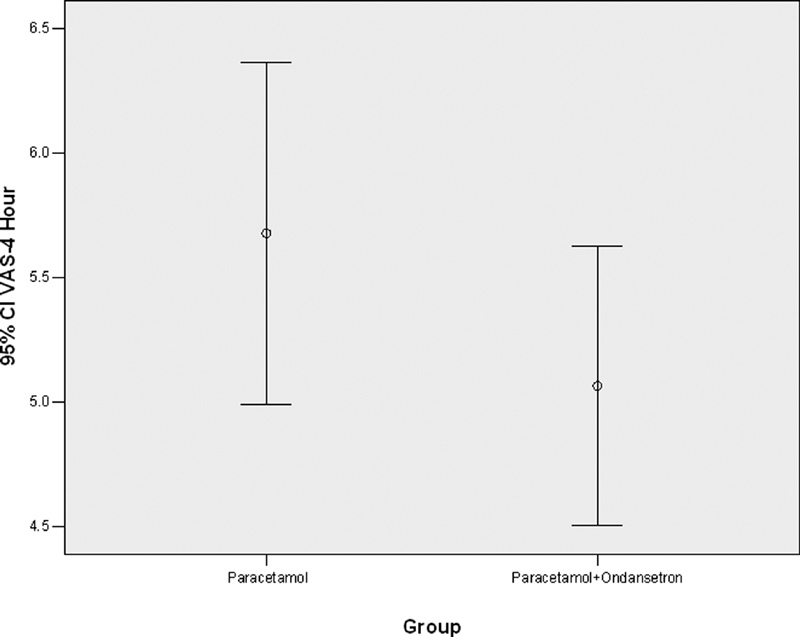
Showing the severity of pain after 4 hours in patients receiving paracetamol alone and those who received paracetamol with ondansetron.

**Fig. 5:**
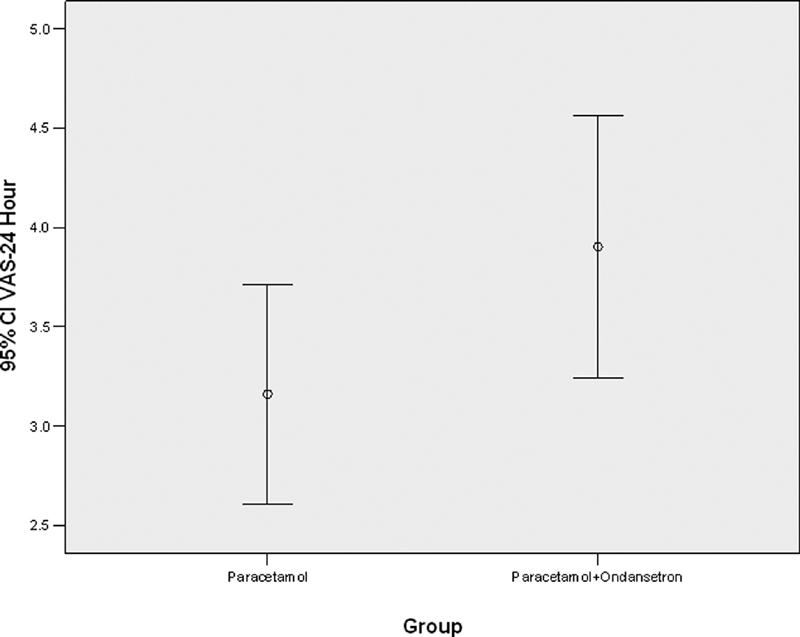
Showing the severity of pain after 24 hours in patients receiving paracetamol alone and those who received paracetamol with ondansetron.

The severity of pain at the time of recovery was similar in two groups ( *p* = 0.653). This similarity is shown in Fig. 3 . Furthermore, the severity of pain after 4 hours was not statistically significant between the two groups ( *p* = 0.162), as is shown in Fig. 4 . Also, the severity of pain after 24 hours in the two groups was not statistically significant ( *p* = 0.083), which is depicted in Fig. 5 .

## Discussion

Nowadays, there are several treatments to reduce pain after surgery, each with its own effectiveness and success. 16 , 17 Opioids are dose-dependent, with side effects such as nausea and vomiting, ileus, itching, urinary retention, respiratory depression, and consequently increased hospitalization time. 18 But the use of nonopioid analgesics reduces side effects. 19 Long-term administration with high doses of analgesics may cause bleeding at the site of gastrointestinal bleeding and renal failure, and nonopioid analgesics do not have these complications. 20 Accordingly, in this study, we compared the effect of paracetamol alone or paracetamol with ondansetron on acute pain after abdominal surgery in hospitals of Islamic Azad University in 2016 to 2017.

The results of this study showed that the severity of postoperative pain in recovery and at 4 and 24 hours after surgery in patients did not differ significantly between patients receiving paracetamol alone and those who received paracetamol with ondansetron. Also, the number of opioid consumed during the operation and in the postoperative phase were similar in patients receiving paracetamol alone and those who received paracetamol with ondansetron. In an interventional study conducted by Bhosale et al in the United States, 19 32 patients were randomized into two groups of patients receiving paracetamol (4 g intravenous) plus ondansetron and those who received paracetamol (4 mg intravenous) with placebo. At the end of the operation, it was announced that the effect of ondansetron in the mix with paracetamol for controlling postoperative pain was greater than placebo compare with paracetamol. In our study, we have used greater sample size and we found that there was no difference in the effect of ondansetron in the mix with paracetamol.

In another interventional study conducted by Alipour et al in Iran, 20 there were 336 patients in the six groups receiving ondansetron (4 mg intravenous), paracetamol (2 mg/kg intravenous), granisetron (2 mg), lidocaine (40 mg), magnesium sulfate (2 mg), and placebo (5 mL normal saline) before surgery and showed that all drugs had a significant reduction in pain intensity versus placebo; however, granisetron and lidocaine had the most effective outcome. 21 But in our study, the addition of ondansetron did not affect the reduction of pain.

In an experimental study conducted by Minville et al, 22 two different protocols were tested on mouse samples after tibia bone fracture. In the first protocol, the mice were divided into four groups after the fracture of the tibia: intraperitoneal injection of paracetamol 30 mg/kg, paracetamol 50 mg/kg, paracetamol 100 mg/kg, or saline. 23

In the second protocol, paracetamol 100 mg/kg plus saline, paracetamol 100 mg/kg with ondansetron 2 mg/kg or saline alone were injected into the peritoneum. It was observed that the reduction in pain intensity for paracetamol was significantly greater than ondansetron. 24 However, in our study, the efficacy of paracetamol was confirmed; though, ondansetron did not increase this efficacy.

In an interventional study conducted by Memis et al in Turkey, 25 20 patients received paracetamol (1 g intravenous every 6 hours to 24 hours) and 20 placebo (100 mL normal saline intravenous every 6 hours to 24 hours). It was reported that paracetamol has a good analgesic effect in reducing postoperative pain, which has been shown in our study.

## Conclusion

Based on the results of this study and their comparison with other studies conducted in this field, it is concluded that the addition of ondansetron to paracetamol has no more effect on reducing acute pain after abdominal surgery, so using it along with paracetamol is not recommended. At the end, it is recommended that further studies be conducted to confirm the findings of this study with a higher sample size and also to evaluate other methods of pain relief after surgery.
