# Supplementary material for: Paracetamol versus Paracetamol Plus Ondansetron on Acute Postoperative Pain
Source: Surg J (N Y). 2021 Dec 15;7(4):e314–8. doi: 10.1055/s-0041-1735899 (PMC8674094; doi:10.1055/s-0041-1735899)
Supplement: Supplementary file 1 — Supplementary Material [file 10-1055-s-0041-1735899-s1900075oa.pdf]

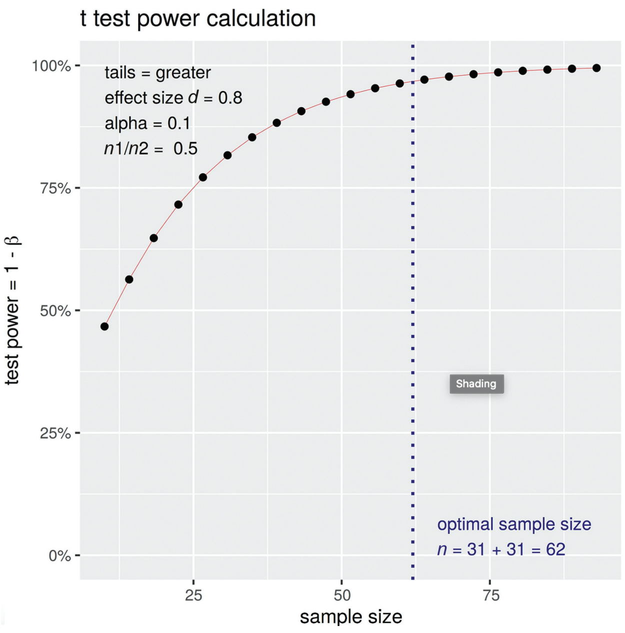

Supplementary Fig. S1 Pain rate in the Paracetamol plus Ondansetron group relative to Paracetamol.

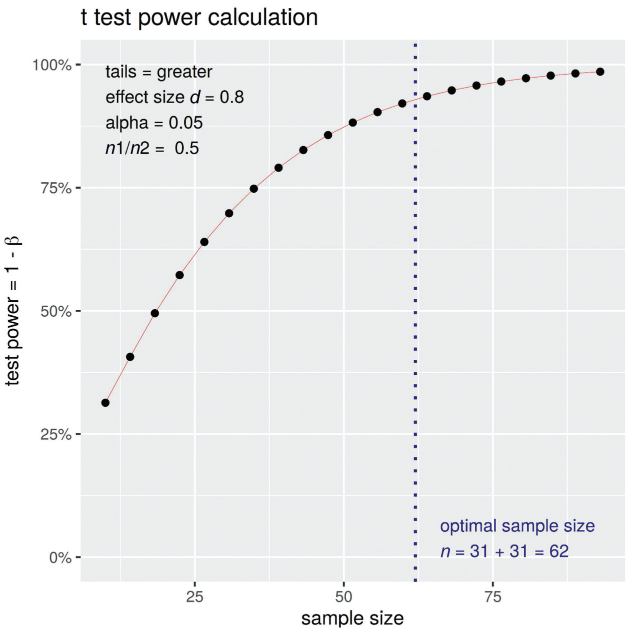

Supplementary Fig. S2 Comparison of pain between two groups with high effect size.

 © 2021. The Author(s).
